# Dorsal Root Ganglia Neurons and Differentiated Adipose-derived Stem Cells: An *In Vitro* Co-culture Model to Study Peripheral Nerve Regeneration

**DOI:** 10.3791/52543

**Published:** 2015-02-26

**Authors:** Alba C. de Luca, Alessandro Faroni, Adam J. Reid

**Affiliations:** ^1^Centre for Neuroprosthesis, EPFL | STI | IMT/IBI | LSBI; ^2^Blond McIndoe Research Laboratories, Institute of Inflammation & Repair, The University of Manchester; ^3^University Hospital of South Manchester

**Keywords:** Neuroscience, Issue 96, Co-culture, neurons, stem cells, neurite outgrowth, peripheral nerve repair, cell-cell interaction

## Abstract

Dorsal root ganglia (DRG) neurons, located in the intervertebral foramina of the spinal column, can be used to create an *in vitro* system facilitating the study of nerve regeneration and myelination. The glial cells of the peripheral nervous system, Schwann cells (SC), are key facilitators of these processes; it is therefore crucial that the interactions of these cellular components are studied together. Direct contact between DRG neurons and glial cells provides additional stimuli sensed by specific membrane receptors, further improving the neuronal response. SC release growth factors and proteins in the culture medium, which enhance neuron survival and stimulate neurite sprouting and extension. However, SC require long proliferation time to be used for tissue engineering applications and the sacrifice of an healthy nerve for their sourcing. Adipose-derived stem cells (ASC) differentiated into SC phenotype are a valid alternative to SC for the set-up of a co-culture model with DRG neurons to study nerve regeneration. The present work presents a detailed and reproducible step-by-step protocol to harvest both DRG neurons and ASC from adult rats; to differentiate ASC towards a SC phenotype; and combines the two cell types in a direct co-culture system to investigate the interplay between neurons and SC in the peripheral nervous system. This tool has great potential in the optimization of tissue-engineered constructs for peripheral nerve repair.

**Figure:**
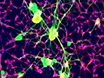


## Introduction

Peripheral nerve injuries are common with approximately 9,000 cases in the UK occurring each year in a predominantly young and working population^1^. Despite microsurgical nerve repair techniques, normal restoration of function is unattainable with resulting impaired hand sensation, reduced motor function and frequent pain and cold intolerance^2^. Such injuries have a profound and permanent impact on the patient and their ability to perform activities of daily living, with less than 60 % returning to work^3^.

Following injury, the phenotype and the morphology of neurons and Schwann cells (SC) change in order to create a suitable environment to allow the axon sprouting. In case of transection, the nerve is divided into proximal and distal stumps; the proximal stump being the point from which the regenerative process takes place, whilst the distal stump undergoes Wallerian degeneration whereupon the SC detach from the injured axons, de-differentiate and proliferate. This is fundamental towards removing myelin debris and preparing the distal stump for nerve re-generation ^4,5^. Axon sprouting is supported by the production of neurotrophic factors and chemokines released by SC at the distal stump, and guided by the basal lamina left behind following Wallerian degeneration^6,7^. SC align alongside the regenerating axon forming the bands of Büngner, which aid the axon growth towards the target organ, reducing branching outside the endoneurial tube. Following reinnervation, SC form the new myelin sheath wrapping the regenerated axons, but sensory and motor function is only partially restored^8^.

Dorsal root ganglia (DRG) are structures located in the intervertebral foramina of the spinal column, containing the sensory neuronal cells innervating the peripheral organs. When dissociated, they can be used as a suitable *in vitro* model for the study of nerve regeneration^9^^–^^11^, including investigations of myelin formation. In particular, adult DRG neurons mimic the *in vivo* characteristics of these cells and provide a formidable tool to study new strategies for peripheral nerve repair in tissue engineering.

Co-cultures represent a dynamic system that simulates *in vitro* the interplay of two (or more) cell types in a particular *in vivo* environment. One of the advantages of these cell co-culture models is the flexibility and high control that can be exerted on the extracellular environment. DRG neurons have been used frequently in co-culture systems with SC to mimic the actual interactions that occur between the two cell types in the peripheral nervous system^10,12^^–^^14^. It was demonstrated that SC secrete extracellular matrix (ECM) proteins and growth factors that can remarkably improve the ability of DRG neurons to survive and sprout neurites^15,16^. However, SC require lengthy periods of time to proliferate and, despite the advances in cell culture technique, it is still hard to generate a suitable number of cells for tissue engineering applications. In addition, the sacrifice of a healthy nerve is necessitated to harvest autologous SC. Therefore, the difference in sourcing SC is important for both tissue engineering and in vitro testing of nerve regeneration. In this view, ASC can be considered a valuable alternative for the development of a tissue-engineered construct to be used for peripheral nerve repair^17,18^. Previous work demonstrated the ability of these cells to differentiate into SC-like ASC, expressing characteristic glial-markers, such as S-100, p75 and glial fibrillary acidic protein (GFAP)^19^, as well as the myelin protein zero (P0)^20^. The secretion of glial growth factors, such as brain-derived neurotrophic factor (BDNF), nerve growth factor (NGF) and glial cell-derived neurotrophic factor (GDNF) was also observed^21,22^. Therefore, SC-like ASC can be used as promoter of peripheral nerve regeneration, as demonstrated by both *in vitro *and *in vivo* studies^23^^–^^26^. In addition, ASC can be harvested through minimally-invasive procedures in higher number compared to other stem cell types; the frequency of stem cells in adipose tissue is 100- to 1,000-fold higher than in bone marrow^27^,and they have a higher proliferation rate compared to SC and bone marrow mesenchymal stem cells.

This work aims to provide a detailed protocol to perform high efficient harvests of dissociated DRG neurons and ASC respectively, the latter being differentiated into SC-like cells. The co-culture of these two cell types will therefore provide a very practical system that can be used for future studies on the ability of DRG neurons to sprout neurites and the mechanisms of myelin formation on different scaffold for nerve tissue engineering.

## Protocol

NOTE: All the experiments involving animals were carried out in accordance with the U.K. Animals (Scientific Procedures) Act, 1986.

### 1. Experimental Set-up

1. Prior to start tissue and cell harvest, check that all the tools are sterile. Whether necessary, autoclave a pair of sharp surgical scissors, a very fine forceps and a fine standard forceps. Also sterilize each substrate before cell seeding using UV sterilization, ethanol exposure or steam sterilization as appropriate.
2. Preparation of media for stem cell harvest and differentiation
  1. Prepare the *stem cell growth medium*, containing Minimum Essential Medium (α-MEM) supplemented with 10 % fetal bovine serum (FBS), 200 mM L-glutamine, and 1 % penicillin-streptomycin (PS).
  2. Prepare a 10 mM stock solution of forskolin by dissolving 10 mg of forskolin in 2.436 ml of sterile dimethyl sulfoxyde. Use at final concentration of 14 μM.
  3. Prepare a 35 mg/ml stock solution of retinoic acid by dissolving 50 mg in 1.43 ml of sterile dimethyl sulfoxyde. Use at final concentration of 350 ng/ml.
  4. Prepare platelet-derived growth factor (PDGF) stocks (100 μg/ml) by dissolving 10 μg of lyophilized powder in 100 μl of distilled sterile water. Use at final concentration of 5 ng/ml.
  5. Prepare basic fibroblast growth factor (bFGF) stocks (100 μg/ml) by dissolving 50 μg of lyophilized powder in 500 μl of distilled sterile water. Use at final concentration of 10 ng/ml.
  6. Prepare the *stem cell differentiation medium*, containing stem cell growth medium supplemented with 14 µM forskolin, 126 ng/ml glial growth factor-2 (GGF-2), 5 ng/mL platelet-derived growth factor (PDGF), and 10 ng/ml basic fibroblast growth factor (bFGF).
3. Preparation of media and stock solutions for neuron harvest and dissociation
  1. Prepare *Bottenstein and Sato’s (BS) medium*^28^, by adding 1 % PS and 1 % N2 supplement to Ham’s F12 medium. Calculate the final volume needed as 500 µl per well (if using a 24-well plate).
  2. Prepare collagenase IV stocks in Ham’s F12 medium at the concentration of 1.25 % wt/v. Filter-sterilize the solution and store at -20 °C as 200 µl aliquots.
  3. Prepare bovine pancreatic trypsin stocks in Ham’s F12 medium at the concentration of 2.5 % wt/v, filter-sterilize and store at -20 °C as 200 µl aliquots.
  4. Prepare the nerve growth factor (NGF) stock solution at the concentration of 5 µg/mL in a filter-sterilized 1 mg/ml fatty acid-free bovine serum albumin (BSA) solution in F12 medium and store at -20 C as 200 µl aliquots. Do not filter the NGF after reconstitution.
4. In case no surface modification has been performed, coat coverslips/plates with poly-D-lysine (0.1 mg/ml for 15 min at RT) and/or laminin (2-10 µg/cm^2^ for 2 hr at 37 °C) to support neuron attachment and neurite outgrowth as appropriate.
5. Always warm up media in a water bath at 37 °C before using.

### 2. Adipose-derived Stem Cell (ASC) Harvest and Differentiation into a SC Phenotype

1. ASC harvest from visceral and inguinal fat of adult male Sprague-Dawley rats
  1. Prior to start, prepare a tube with 10-15 ml of Hanks’ Balanced Saline Solution (HBSS) supplemented with 1% v/v of PS solution and store on ice until fat harvesting.
  2. Terminate the rat by cervical dislocation and decapitation. Shave the rat and make an incision through the abdominal skin, exposing the internal organs and avoiding bleeding. Remove the visceral fat encasing the stomach and intestines (usually characterized by a fatty yellow consistency) and the inguinal fat surrounding the testicles from an adult male Sprague-Dawley rat. Transfer the fat in the tube containing HBSS on ice.
  3. In a biological safety cabinet (class II) finely chop the fat using a pair of scissors and a sterile razor blade until fine consistency is reached and transfer it into a tube containing 15 ml of 0.2 % wt/v collagenase type I solution freshly prepared and filter-sterilized on the day.
  4. Transfer the tube in a water bath at 37 °C and leave the fat tissue to digest in presence of the enzyme for 30 min-1 hr under continuous agitation. Monitor the digestion closely and stop before the tissue is fully dissociated, this will improve cell viability and cell yield. Filter theun-dissociated tissue through a 100 µm cell strainer. NOTE: Good tissue digestion will result in homogenous consistency of the fat, visible by eye when gently swirling the tube, acquiring a beige appearance.
  5. Neutralize the enzyme by adding 15 ml of stem cell growth medium containing fetal bovine serum at 37 °C and centrifuge the solution at 160 g for 10 min in order to collect the stromal vascular fraction, including the stem cells, at the bottom of the tube.
  6. At this stage, the pellet can be resuspended in 1 ml of red blood cells lysis buffer to remove blood cell contamination. After resuspension and pipetting for 1 min, add 10 ml of fresh stem cell growth medium and centrifuge at 160 g for 10 min.
  7. Carefully aspirate the supernatant from the tube, taking care of the deposited cell pellet at the bottom. Resuspend the cells in 10 ml of stem cell growth medium, transfer them into a 75 cm^2^ flasks and incubate at 37 °C, 5 % CO_2_. Maintain the cells at sub-confluent levels until passage 1-2, changing medium every 3 days.
2. ASC differentiation to a SC phenotype
  1. At passage 1-2, remove the stem cell growth medium from the 75 cm^2^ flask and replace it with 10 ml of fresh medium containing 1 mM β-mercaptoethanol freshly prepared and filter-sterilized on the day. Incubate the cells at 37 °C, 5 % CO_2_ for 24 hr. At this stage, it is important that the cells are seeded at low density (30 %) before starting the differentiation.
  2. Wash the cells carefully with HBSS, aspirate and replace it with 10 ml of medium containing 350 ng/ml retinoic acid. Incubate the cells at 37 °C, 5 % CO_2_ for 72 hr. Try to minimize exposure of cell medium to light.
  3. After 3 days, wash the cells carefully with HBSS, aspirate and replace it with 10 ml of stem cell differentiation medium (see step 1.2.6). Maintain the cells at sub-confluent levels, changing medium every 3 days. NOTE: Following 2 weeks of incubation, ASC are differentiated into SC-like ASC, expressing their characteristic phenotype (as demonstrated by Kingham *et al.*^5^). They can then be used up until the 10th passage without noticeable changes in behavior^29^ .

### 3. Harvest and Dissociation of Dorsal Root Ganglia (DRG) Neurons

1. Terminate the rat by cervical dislocation and decapitation. Shave the rat and lift the skin to expose the spinal column. Using sharp scissors, excide the spinal column taking extra care of the confined organs and blood vessels. Transfer the spinal column in a biological safety cabinet (class II) using a Petri dish and remove any dorsal part.
2. Divide the spinal column in half along the longitudinal axis using sterile and sharp surgical scissors to expose the cord tissue. At this point, it is helpful to cut the spinal column in two smaller segments below the level of the rib cage, to make it easier to handle during the DRG neuron harvest. Using fine forceps, gently remove all the cord tissue, paying attention to not pull and remove the DRG roots. This way the DRG and roots will be exposed within the vertebral canals, still encased in the column. Observe DRG as white filaments coming out directly from the canals.
3. Pull out the entire DRG root (not just the DRG) from the vertebral canals by using very fine forceps, going deep into the vertebral canals and paying attention not to damage the roots of the ganglia. Transfer the DRG into a small petri dish (60 mm^2^) containing 3-4 ml of Ham’s F12 medium supplemented with 1% PS. If using different animals, use separate dishes.
4. Under a dissecting microscope, clean the DRG of any excess of nerve roots surrounding the ganglia using sterile forceps and a scalpel to reduce glial cell contamination. Transfer the DRG into a small petri dish (35 mm^2^) with 1.8 ml of fresh F12 medium.
5. Add 200 µl of 1.25 % wt/v collagenase type IV stock solution (final concentration of 0.125%) and incubate the DRG at 37 °C, 5 % CO_2_ for 1 hr. Carefully aspirate the medium with a glass pipette, paying attention not to aspirate or damage the DRG. Add fresh F12 medium supplemented with 0.125 % wt/v collagenase type IV enzyme and incubate for 1 hr as previously described.
6. Aspirate the medium and gently wash the DRG with F12 medium. Add then 1.8 ml of F12 medium and 200 µl of trypsin (final concentration of 0.25 % wt/v) and incubate at 37 °C, 5 % CO_2_ for 30 min.
7. Remove trypsin and add 1 ml of F12 medium supplemented with 500 µl of FBS to arrest the enzymatic reaction. Aspirate the medium and gently wash the DRG with F12 medium for three times to remove traces of serum.
8. Add 2 ml of fresh F12 medium and carefully transfer the DRG with the medium into a 15 ml tube using a glass pipette. Gently dissociate the DRG neurons by pipetting up and down (about 8-10 times) with the glass pipette (plastic tips can be used as alternative).
9. Allow the pellet to settle at the bottom of the tube and collect the medium in a new tube. Add 2 ml of fresh F12 medium to the tube containing the pellet and repeat the mechanical dissociation with the glass pipette. Repeat this step until the suspension becomes homogeneous (about 3-4 times) and collect all the dissociated DRG in the new tube. This method reduces the stress deriving from the mechanical dissociation and improves the viability of the neurons.
10. Filter the resulting homogenized suspension into a new 15 ml tube using a 100 µm cell strainer to remove un-dissociated neurons and other debris. At this stage, it might be convenient to first filter the cell suspension into a 50 ml tube and then transfer the solution into the smaller tube using a glass pipette. Centrifuge the suspension at 110 g for 5 min.
11. Prepare a 15 % bovine serum albumin (BSA) by adding 500 µl of a 30 % BSA solution to 500 µl of F12 medium. Slowly pipette the solution down the wall of a 15 ml tube to create a gradual protein trail. At this stage, it is helpful to hold the tube at a 45° angle and slowly release the BSA using the numbers on the falcon tube as a reference for the forming “track”.
12. Aspirate the supernatant from step 3.10 leaving 500 µl at the bottom of the tube and resuspend the cell pellet in the same medium. Slowly pipette the suspension along the protein trail previously prepared in step 3.11 (use the numbers on the tube as a reference) and centrifuge at 500 g for 5 min.
13. Aspirate the supernatant and resuspend the pellet in 1 ml of modified BS medium (or a mixed medium for the SC-like ASC/DRG co-culture, as described below in step 4.5. NOTE: The volume to re-suspend the DRG neurons can be made up to the actual volume required, depending on the final cell concentration desired and the number of samples to be seeded. One animal will provide enough cells for a 24-well plate experiment.
14. Incubate the seeded samples at 37 °C, 5% CO_2_ for 2 hr to allow cell attachment and finally add fresh BS medium supplemented with 50 ng/ml of nerve growth factor (NGF).

### 4. Direct Co-culture of SC-like ASC and DRG Neurons

1. Maintain SC-like ASC in culture at sub-confluent levels as described in step 2.2.3. Twenty-four hr prior to DRG harvest, aspirate the stem cell differentiation medium and wash the cells with HBSS. Aspirate and incubate in 3 ml of trypsin at 37 °C, 5% CO_2_ for 3 min.
2. Check under a light microscope that all the cells have detached from the flask. Gently tap the flask to help detachment. Add 7 ml of medium to stop the trypsin reaction, collect the cell suspension in a 15 ml tube and centrifuge at 110 g for 5 min.
3. Aspirate the supernatant and resuspend the cell pellet in 5 ml of stem cell differentiation medium. Count the cells using a hemocytometer and dilute the cell suspension according to the final concentration required. Ideally, seeding 20,000 SC-like ASC/cm^2^ would guarantee a confluent layer of cells.
4. Seed the SC-like ASC on the substrate and incubate at 37 °C, 5 % CO_2_ for 24 hr to allow cell attachment.
5. After 24 hr incubation, aspirate the medium and add the DRG neurons on top of the cells as described in step 3.14 and 3.15. Change the medium to a mixed medium containing 50 % of stem cell differentiation medium and 50 % of BS medium. In addition, reducing the FBS concentration to 1-2.5 % in the final mixed medium helps avoiding the proliferation of contaminating satellite cells derived from the DRG dissociation.
6. Incubate the co-cultured samples at 37 °C, 5 % CO_2_ and maintain in culture for the required time for future tests.

## Representative Results

Cultures of dissociated DRG neurons represent a suitable *in vitro* model for the study of nerve regeneration. However, untreated substrates do not provide a suitable environment for DRG attachment and extension of neurites. SC-like ASC are able to produce growth factors and chemokines^19^ which can improve the ability of DRG neurons to sprout neurites when they are released in the culture medium. The presence of SC-like ASC in the culture system can therefore play an important role in regulating the DRG functions.

This protocol (**Figure 1**) illustrates the procedure to perform a direct co-culture of SC-like ASC and DRG neurons, during which neuronal cells get in direct contact with previously seeded SC-like ASC. The main difficulty associated with this procedure is the high variability of the number of DRG neurons harvested from different animals. For this reason, results can be sometimes hard to compare and a particular attention should be paid during the seeding procedure in order to obtain always a comparable cell density in each experiment. The protocol has been optimized to reduce at least the number of satellite cells remaining from the DRG neuron dissociation using the BSA gradient (step 3.11). In addition, cytosine-arabinose (ARA-C) supplement can be added to the BS medium in order to further minimize the satellite cell population, as described by Kingham *et al*.^11^. However, the choice of the treatment time needs to be carefully balanced with the DRG and SC-like ASC vitality, also affected by the presence of ARA-C supplement in the culture medium. Therefore, it is very hard to accomplish a satellite cell free-system.

**Figure 2** shows the importance of SC-like ASC for DRG neurite sprouting in a co-culture model using untreated and chemically-modified poly--caprolactone (PCL) films as substrates. DRG neurons were maintained in culture for 3 days in presence or absence of SC-like ASC, using a mixed solution containing 50 % of BS medium and 50 % of stem cell differentiation medium, as described in step 4.5. Following this period, cells were fixed in 4 % paraformaldehyde and stained with β-tubulin III (DRG neurons) and S100 (SC-like ASC) to investigate cell morphology and to study the ability of DRG neurons to protrude neurites. No neurites were observed on untreated surfaces in absence of SC-like ASC (**Figure 2A**), whilst the formation of neurites was clearly improved in the co-culture system (**Figure 2C**). On average, the number of neurites per cell body significantly increased from 0 to 3 in presence of stem cells. Confirmation of these results was also given by scanning electron microscopy (SEM) analysis, shown in **Figure 3**. In particular, the SEM images show that the ability of DRG neurons to sprout neurites occurs preferentially in conjunction with SC-like ASC, as indicated by the yellow arrows in **Figure 3C**.

It should be pointed out that the use of laminin-modified substrates (including laminin-derived peptides) in culture systems containing DRG neurons has been frequently defined as a suitable condition for neuronal cell culture, having remarkable effects on neurite formation and extension^30^^–^^32^. However, the results shown in **Figure 2D** and **Figure 3D** demonstrates that the combination of chemical and biological cues can further enhance the response of DRG neurons.


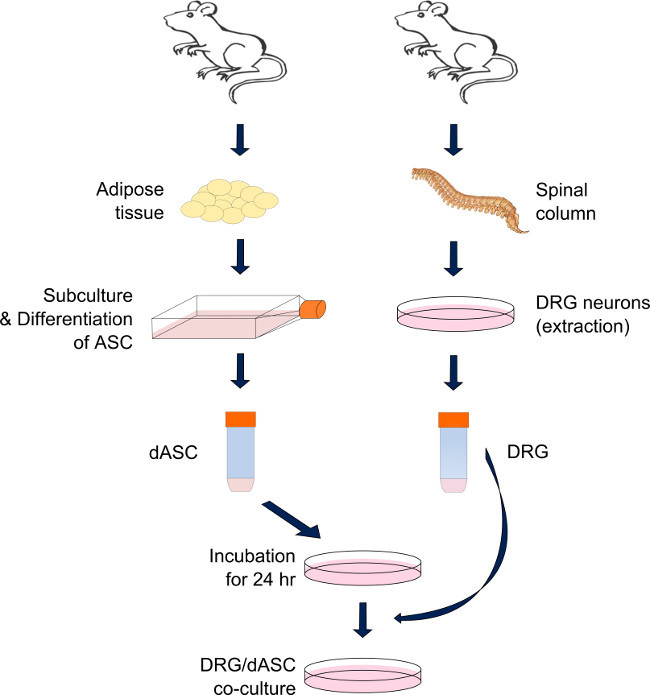
**Figure 1. Preparation of the direct DRG-SC-like ASC co-culture system.** DRG neurons and ASC are derived respectively from the spinal column and the visceral and inguinal fat of adult male Sprague-Dawley rats. After the digestion of the adipose tissue through a cascade of enzymatic reactions, ASC are differentiated into SC-like cells and maintained in sub-confluent conditions until needed. SC-like ASC are pre-seeded on each substrate 24 hr prior the DRG harvest. On the day, DRG neurons are extracted and dissociated through a series of enzymatic and mechanical actions. The neurons are then seeded on the top of the previously seeded SC-like ASC and maintained in culture until assayed (mixed medium: containing 50 % of stem cell differentiation medium and 50 % of modified BS medium). Please click here to view a larger version of this figure.


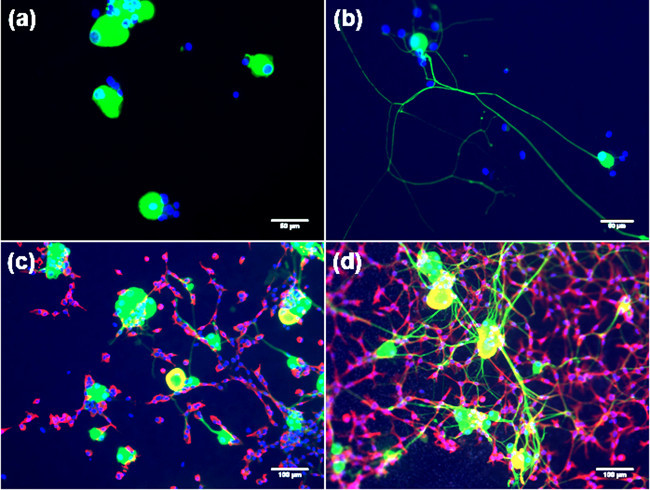
**Figure 2. Fluorescence images of DRG neurons in diverse culture conditions.(A)** untreated PCL films;** (B) **RGD-modified PCL films; **(C)** co-cultured with SC-like ASC on untreated PCL films; **(D)** co-cultured with SC-like ASC on RGD-modified PCL films. Cells were maintained in culture for 3 days using a mixed solution containing 50 % of modified BS medium and 50 % of stem cell differentiation medium. After this time, cells were fixed in 4 % paraformaldehyde, permeabilized in a Triton-X solution, and non-specific binding sites were blocked with 1 % BSA. Neuronal cells were then stained against β-tubulin III (FITC; green) and SC-like ASC against S-100 (AlexaFluor568; red) antibodies. Finally nuclei were stained with DAPI (blue). Images were acquired using a fluorescence microscope (Olympus BX60, Japan). (Re-print with permission from de Luca *et al.*^30^. Please click here to view a larger version of this figure.


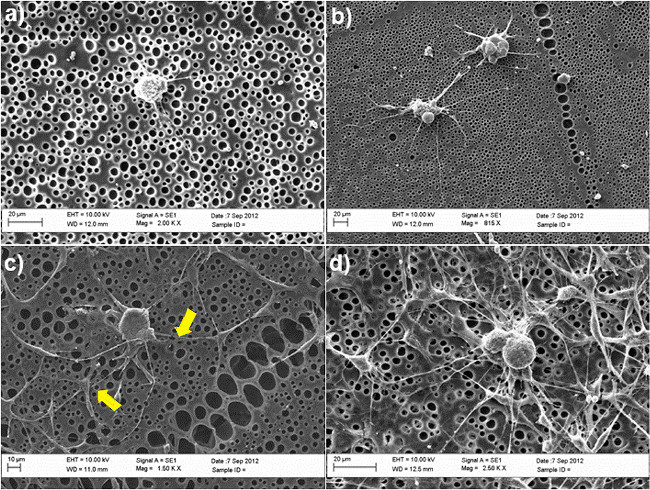
**Figure 3. SEM images of DRG neurons indiverse culture conditions.(A)** untreated PCL films **(B)** RGD-modified PCL films;** (C)** co-cultured with SC-like ASC on untreated PCL films; **(D)** co-cultured with SC-like ASC on RGD-modified PCL films. The yellow arrows indicate the contact points between SC-like ASC and DRG, confirming their direct interaction in the co-culture system. Cells were maintained in culture for 3 days and fixed in 2.5 % glutaraldehyde. Following dehydration in graded ethanol series (50 %, 70 %, 90 %, 100 %), cells were finally rinsed in hexamethyldisilazane and dried before mounting on stubs and gold sputtering for SEM analysis. Images were acquired using a SEM (Zeiss EVO60, UK) with an accelerating voltage of 10kV. (Re-print with permission from de Luca *et al.*^30^. Please click here to view a larger version of this figure.

## Discussion

DRG neurons are frequently used among neuronal cells for primary culture to study the regeneration of neurons after axotomy *in vivo*. Here an accurate protocol for DRG harvest from adult rats is presented, aimed at reducing the population of satellite cells in the surrounding environment without compromising the neuronal survival. As ASC differentiated into a SC-like phenotype are a valid alternative to SC for cell therapies, a SC-like ASC/DRG co-culture system is also described in detail.

It is widely known that laminin (or laminin-derived peptide sequences) have a beneficial effect on neuron survival and neurite formation^31^^–^^33^. When performing DRG neuron cultures it is therefore advised to previously coat each substrate with laminin in order to avoid any loss of functionality of the neuronal cells. The concept of laminin coatings also applies to biomaterial substrates for the design of tissue engineering constructs, such as poly--caprolactone (PCL) frequently used for the fabrication of nerve conduits^30^. Also, previous work demonstrated that fibrin matrices are suitable materials for neuronal cultures in three dimension^11^.

In addition to protein coating, co-culture models provide valuable conditions for DRG neuron survival and a suitable environment to study the interactions that occur between neurons and SC in the peripheral nervous system following injury. Cells can also be transplanted *in vivo *by using neural devices in order to shorten the recruitment time of autologous cells at the injury site. This is particularly important in severe injuries, which can lead to cell senescence/death and muscle atrophy. Although SC are the most important glial cells involved in the process of peripheral nerve regeneration and myelination, their limited availability and their slow proliferation rate makes them unsuitable for tissue engineering applications^34^. ASC are a valid alternative due to their abundance and ability to differentiate into the SC phenotype, expressing specific glial markers and showing functional similarities to native SC^19^. ASC are also able to produce proteins and growth factors^5^ that can be beneficial to the formation and extension of neurites by DRG neurons in a co-culture system. However, two different co-culture systems can be set up as function of the experimental needs. The method proposed in this paper is a revised version of a well-established protocol in our laboratory^11 ^and it involves a direct contact between the two cell types (*direct co-culture*), in which the second cell type (DRG neurons) are seeded on the top of the others (SC-like ASC). This approach is based on previous findings that demonstrated the importance of the presence of a glial cell layer on the substrate when seeding neuronal cultures^35^^–^^37^. This effect is likely due to cell-cell interactions through DRG integrins and ECM molecules deposited from ASC and other cues on the stem cell surface. It was also observed that a reduction of protein serum in the medium reduced the proliferation of contaminating satellite cells that can derive from the dissociation of DRG neurons, without affecting ASC functions. However, satellite cells, including a small population of SC, are hard to completely eliminate from cultures of DRG neurons and few remaining cells will also be present in the co-culture system. It is therefore important to note that these small sub-populations can also participate to myelination processes during *in vitro* studies, recalling the autologous cells that are present *in vivo* after injury. The second approach (not presented here) involves the use of cell culture inserts, avoiding a direct contact between the two different cell types (*indirect co-culture*). However, it is not representative of the *in vivo* conditions during nerve regeneration (reduced ability of neuronal cells to develop long neurites), but it is used to investigate the effect of released diffusible factors in the medium by a certain cell population onto the other^38^.

## Disclosures

The authors confirm that there are no conflicts of interest associated with this publication.
